# B-cell lymphoma-extra large expression correlated with protein kinase RNA-like ER kinase and ubiquitination signals in the type 1 diabetes mouse model

**DOI:** 10.1097/PR9.0000000000001356

**Published:** 2025-11-11

**Authors:** Yu-Yu Kan, Ying-Shuang Chang, Tzu-Ning Chao, Yu-Lin Hsieh

**Affiliations:** a School of Medicine, College of Medicine, National Sun Yat-sen University, Kaohsiung, Taiwan; b Department of Anatomy, School of Medicine, College of Medicine, Kaohsiung Medical University, Kaohsiung, Taiwan; c School of Post-Baccalaureate Medicine, College of Medicine, Kaohsiung Medical University, Kaohsiung, Taiwan; d Department of Medical Research, Kaohsiung Medical University Hospital, Kaohsiung, Taiwan

**Keywords:** Diabetic neuropathy, ER stress, B-cell lymphoma-extra large, Protein kinase RNA-like ER kinase, Eukaryotic initiation factor 2α, Ubiquitin D

## Abstract

**Introduction:**

Diabetes mellitus with pain sensation is referred to as diabetic painful neuropathy (DN). DN development results from endoplasmic reticulum (ER) stress, including the upregulation of ER stress signaling–related molecules and autophagy. B-cell lymphoma-extra large (Bcl-_XL_), a protein of the antiapoptotic Bcl2 family, exhibits neuroprotective efficacy and expression profiles similar to those of protein kinase RNA-like ER kinase (PERK) in DN.

**Objectives:**

To investigate the roles of Bcl-_XL_ in type 1 diabetes mellitus (T1DM).

**Methods:**

This report used a single dose of streptozotocin (200 mg/kg) to establish a mouse model of T1DM, and to assay the changes in the expression profile of Bcl-_XL_ and ER stress-related signal molecules with the dorsal root ganglia tissues.

**Results:**

This study discovered the coexpression of Bcl-_XL_ upregulation and PERK, a downstream mediator of ER stress (M1 Manders coefficients: 0.48–0.61, M2 Manders coefficients: 0.41–0.61). PERK phosphorylated eukaryotic initiation factor 2α (p-eIF2α) in the same sensory nociceptors (M1 Manders coefficients: 0.94–0.98, and M2 Manders coefficients: 0.97–0.98). This eIF2α upregulation was inversely correlated with thermal latencies (*r* = –0.89, *p* < 0.0001) and mechanical thresholds (*r* = –0.75, *p* < 0.0001). In addition to PERK upregulation, ubiquitin D (UBD), a key molecule in ubiquitination signaling, was upregulated and inversely correlated with neuropathic pain in DN.

**Conclusions:**

This study uncovers a new neuropathology underlying T1DM-associated DN: Bcl-_XL_ activation associates PERK and ubiquitination marker upregulation in sensory neurons. Bcl-_XL_ inactivation in nociceptors might be a potential target for relieving neuropathic pain of T1DM.

## Abstract 2

B-cell lymphoma-extra large activation associates endoplasmic reticulum stress and ubiquitination signaling in type 1 diabetes mellitus. Targeting B-cell lymphoma-extra large is a new direction for diabetic neuropathy therapy.

Supplemental Digital Content is Available in the Text.

## 1. Introduction

Chronic type 1 diabetes mellitus (T1DM) leads to the development of complicated metabolic disorders, endoplasmic reticulum (ER) stress–associated autophagy, and diabetic painful neuropathy (DN).^11,19,21^ The pathological mechanisms of DN remain unclear, and currently available therapies for DN are unsatisfactory.^8,22,35,36^ Diabetic neuropathy development is the most prevalent complication of DM, affecting approximately 50% of patients with DM. Particularly, patients with T1DM experience pain, tingling, and numbness sensations in the limbs in 57% of cases in the chronic stage of T1DM.^9^ Studies have demonstrated that the activation of protein kinase C epsilon (PKCε), an important intracellular signaling molecule in primary afferent nociceptors, mediates ER stress–associated autophagy.^10,21^ Protein kinase C epsilon was also coexpressed with B-cell lymphoma-extra large (Bcl-_XL_), a protein of the antiapoptotic B-cell lymphoma 2 (Bcl2) family.^10^ B-cell lymphoma-extra large has neuroprotective properties, which are evoked in response to cellular stress, because it regulates cellular energy homeostasis.^13,17^ Accordingly, Bcl-_XL_ might be a signaling molecule that is activated under nerve injury and that is correlated with ER stress.

Protein folding, modification, and trafficking occur in the ER, and ER stress is a protective mechanism for eliminating misfolded proteins. Many studies have examined the expression profiles of functional proteins involved in ER stress in a range of neurological disorders, particularly in peripheral neuropathy.^14,15,28,30,32,34^ Intracellular signaling molecules mediate ER stress. For example, the phosphorylation of protein kinase RNA-like ER kinase (PERK) mediates ER stress, which sequentially phosphorylates eukaryotic initiation factor 2α (eIF2α), followed by downstream gene transcription. Pathogenic misfolded proteins in the ER are retrotranslocated into the cytoplasm for further protein degradation procedures; for example, the dynamic balance of ubiquitination, deubiquitination, and degradation of these target misfolded proteins is maintained by ubiquitin-proteasome system (UPS),^26^ including ubiquitin D (UBD). Ubiquitin D is a key molecule mediating ubiquitination signaling involved in protein degradation. The coexpression profiles of UBD:PERK might be correlated with DN.

In this study, our previously established DN model^10,19^ was used to assay the changes in the expression profile of Bcl-_XL_ and the coexpression profile of PERK and UBD in dorsal root ganglia (DRG). The present study discovered that the upregulation of Bcl-_XL_(+):PERK(+), PERK(+):eIF2α(+), and PERK(+):UBD(+) neuron densities was correlated with the development of neuropathic pain. These neuron densities were also inversely correlated with thermal latencies and mechanical thresholds. The results jointly indicate that Bcl-_XL_ expression is associated with PERK and ubiquitination signaling upregulation. Thus, inactivating Bcl-_XL_ might be a treatment strategy for neuropathic pain in T1DM.

## 2. Materials and methods

In this study, a T1DM mouse model was established through the intraperitoneal injection of streptozotocin (200 mg/kg; Sigma, St. Louis, MO) into 8-week-old adult male C57/B6 mice, because male diabetic individuals are susceptible to nerve damage^5,7^ and are more likely to experience an early onset of peripheral neuropathy.^1^ The blood glucose level was assessed weekly using a commercially available glucometer ACCU-CHEK GO (Roche Diagnostics GmbH, Mannheim, Germany). Mice showing severe hyperglycemia (blood glucose >400 mg/dL) were assigned to the DN group, and mice showing mild to moderate hyperglycemia (blood glucose <400 mg/dL) were assigned to the non-DN (nDN) group. Mice also received citrate solution (pH = 5.5, Sigma), serving as the control group (the citrate group). Mice were housed in plastic cages under a 12-hour light/12-hour dark cycle, with ad libitum access to water and food. The results of all the experiments, including behavioral tests and immunofluorescence assay, were examined at 2 months after DN induction (DNm2) and 5 months after DN induction (DNm5). All procedures were performed in a blinded and coded manner. In addition, the study protocol was conducted following the ethical guidelines for laboratory animals,^37^ and efforts were undertaken to minimize animal suffering. The Institutional Animal Care and Use Committee of Kaohsiung Medical University approved the study protocol (approval no. 110115).

### 2.1. Evaluation of neuropathic pain

Before the evaluations, the activity and appearance of mice were assessed by determining their thermal (hot plate test) and mechanical (von Frey monofilament test) responses.

#### 2.1.1. Hot plate test

Mice were placed on a 52°C hot plate (IITC, Woodland Hills, CA) enclosed in a Plexiglas cage. The withdrawal response from the hot plate was as follows: (1) shaking, (2) licking, or (3) jumping, and the withdrawal latency of the hind paw to the thermal stimulation was determined to an accuracy of 0.1 second. There were 3 trials for each mouse that were conducted at 30-minute intervals, and the thermal latency was represented for each mouse as the average of 3 trials of thermal stimulation.

#### 2.1.2. von Frey monofilament test

The changes in the mechanical threshold of the mice in each group were assessed using the up-down method with various calibers of von Frey monofilaments (Somedic Sales AB, Hörby, Sweden) and following our established protocol.^20,23^ In brief, a series of monofilaments was applied to the plantar region of the hind paw of each mouse. If paw withdrawal occurred, a monofilament of a smaller caliber was applied. If the paw was not withdrawn, a monofilament of a larger caliber was applied. Four additional stimuli with monofilaments of various calibers were applied based on the preceding responses of the mice, and the mechanical thresholds were calculated using an established formula.^12,19^ There were 2 trials for each hind paw of the mouse (a total of 4 trials) that were conducted at 20-minute intervals, and the mechanical thresholds of each mouse were calculated by the mean of 4 times of monofilament applications.

### 2.2. Immunofluorescence staining of dorsal root ganglia tissues

For this study, cryostat sections with a thickness of 8 µm were obtained from lumbar DRG. For systematic sampling, 2 ganglia (L4 and L5) per mouse were collected for sectioning. Every section of each ganglion, separated by an interval of 80 µm, was immunostained and quantified. Conventional double-labeling immunostaining was performed for the sections. In brief, the sections were incubated overnight at 4°C with a combination of primary antisera. Thereafter, the sections were incubated with the corresponding secondary antisera for 1 hour. The primary antisera included the anti-Bcl-_XL_ (rabbit, 1:250; Cell Signaling, Danvers, MA), anti-PERK (goat, 1:150; R&D Systems, Minneapolis, MN), anti-eIFα (rabbit, 1:600; Thermo Fisher Scientific, Waltham, MA), and anti-UBD (rabbit, 1:200; Proteintech, Rosemont, IL) antisera. We employed the following combinations of primary antisera: (1) Bcl-_XL_–PERK, (2) PERK–eIF2α, and (3) PERK–UBD. The antisera used in this study were validated for specificity by negative immunostaining control (Supplementary Figure 1, available at http://links.lww.com/PR9/A353). The sections with immunofluorescence staining were systematically photographed at 200× under a fluorescence microscope (Axiophot microscope, Carl Zeiss, Heidelberg, Germany). Thus, a montage of the entire DRG was created per established procedures.^19^ Dorsal root ganglia tissues are composed of different types of cells, and the ganglia neurons were larger than other cell types, such as fibroblasts, and only neurons with a clear nuclear profile were counted. The optical intensity between immunoreactive and background neurons were determined. On a scale of 0 to 255, a preliminary analysis revealed that the optical intensities of Fluorescenin isothiocyanate were in the range of 138 to 255. Similarly, the optical intensities of Texas red (TR) were in the range of 111 to 248. Each signal of a fluorochrome with optical intensity below these ranges was defined as the background. The colocalized proportion was estimated by using the Manders coefficient to express the fraction of fluorescence intensity in a specific channel (ImageJ version 2.1.0, National Institutes of Health, Bethesda, MD). Neuron density is expressed as neurons/mm^2^.

### 2.3. Statistical analysis

To minimize individual bias, each time point of the experimental approach included at least 5 animals. Moreover, grouping information was blinded during behavioral tests and all quantification procedures. All data are expressed as mean ± SD of the mean. A one-way repeated-measures analysis of variance was performed, followed by Tukey post hoc test, with *P* < 0.05 indicating statistical significance.

## 3. Results

### 3.1. B-cell lymphoma-extra large and protein kinase RNA-like ER kinase colocalization in dorsal root ganglia with streptozotocin-induced chronic neuropathic pain

**Figure 1.**
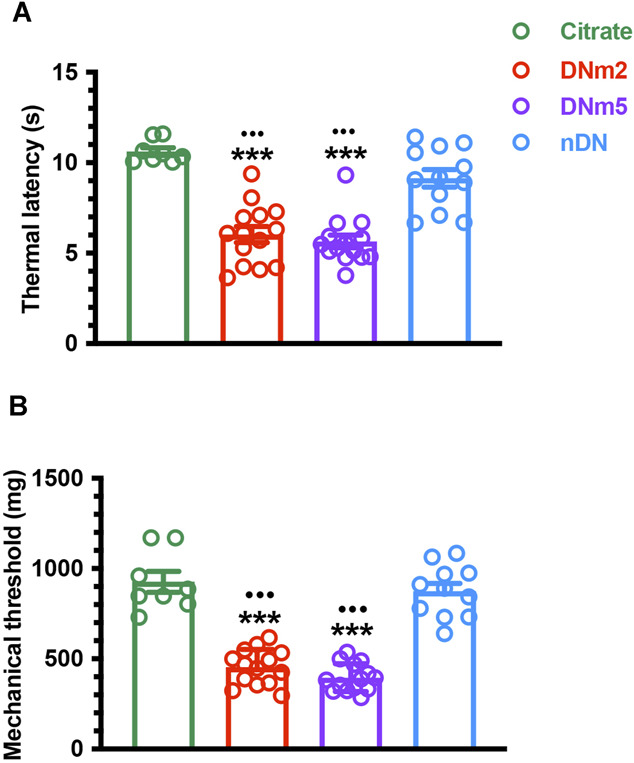
Development of neuropathic pain in mice with streptozotocin (STZ)-induced diabetic neuropathy (DN). (A, B) A mouse model of DN was established through the intraperitoneal injection of STZ (200 mg/kg), and neuropathic pain was evaluated using the hot plate test (A) and von Frey monofilament test (B) in the citrate (n = 8) and DN groups (blood glucose >400 mg/dL) at 2 months after DN (DNm2, n = 14) and 5 months after DN (DNm5, n = 14) and in the non-DN (nDN) group (blood glucose <400 mg/dL, n = 12). At DNm2 and DNm5, mice changed the thermal latencies and mechanical thresholds. Each group is labeled with a color bar indicated in A and B. (A, B) Changes in thermal latency (A) and mechanical threshold (B). ***P < 0.001: DNm2 or DNm5 vs the citrate group. •••*P* < 0.001: DNm2 or DNm5 vs the nDN group.

**Figure 2.**
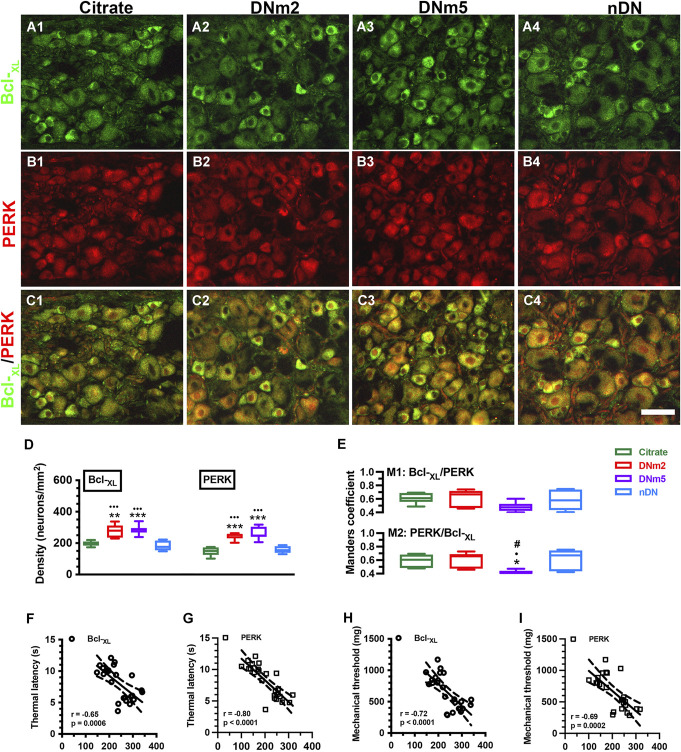
Neuromorphological examinations for B-cell lymphoma-extra large (Bcl-_XL_)(+): protein kinase RNA-like ER kinase (PERK)(+) neurons in mice with streptozotocin (STZ)-induced diabetic neuropathy (DN). Double-labeling immunofluorescence staining was performed with anti-Bcl-_XL_ (A1–A4, green) and anti-PERK (B1–B4, red) antiserum in the dorsal root ganglion in the citrate group (A1, B1, and C1; n = 6), DNm2 (A2, B2, and C2; n = 6), DNm5 (A3, B3, and C3; n = 7), and non-DN (nDN) groups (blood glucose <400 mg/dL; A4, B4, and C4; n = 5). (C) Photographs of Bcl-_XL_(+):PERK(+) neurons (A3–E3) are merged for the Manders coefficient colocalization. DNm2 and DNm5: mice with blood glucose >400 mg/dL at 2 and 5 months after receiving STZ, respectively. Bar, 50 µm. (D, E) Density changes in Bcl-_XL_(+) (left panel in D), PERK(+) (right panel in D), and (E) Manders coefficients M1 (upper panel) and M2 (lower panel) of Bcl-_XL_(+):PERK(+) neurons according to colocalized patterns in C1–C4. Bcl-_XL_(+):PERK(+) neurons were upregulated at DNm2 and DNm5. *P < 0.05, ***P < 0.001: DNm2 or DNm5 vs the citrate group. •*P* < 0.05, •••*P* < 0.001: DNm2 or DNm5 vs the nDN group. #*P* < 0.05: DNm2 vs the DNm5 group. (F–I) Densities of Bcl-_XL_(+):PERK(+) neurons are inversely correlated with changes in thermal latency (F, G) and mechanical threshold (H, I).

In the DN group, thermal latencies (6.0 ± 1.7 vs 10.6 ± 0.6 seconds, *F* = 29.79, *P* < 0.0001) and mechanical thresholds (453.1 ± 98.4 vs 926.4 ± 163.6 mg, *F* = 61.27, *P* < 0.0001) were changed at DNm2 and DNm5 (thermal latency: 5.6 ± 1.3 seconds, *P* < 0.0001, and mechanical thresholds: 395.4 ± 77.0 mg, *P* < 0.0001). Comparatively, these thresholds were higher in the citrate and nDN groups (thermal latency: 9.1 ± 1.7 seconds, *P* < 0.0001, and mechanical thresholds: 874.4 ± 144.3 mg, *P* < 0.0001) (Fig. 1A and B). In neuromorphological examinations, Bcl-_XL_ is mainly expressed by small-sized sensory neurons, and a high proportion of Bcl-_XL_(+) neurons was found, and these Bcl-_XL_(+) neurons were upregulated at DNm2 (277.7 ± 42.4 vs 197.1 ± 16.0 neurons/mm^2^, *F* = 16.43, *P* = 0.0015) and DNm5 (284.7 ± 31.1 neurons/mm^2^, *P* = 0.0004) compared with the citrate and nDN groups (183.3 ± 32.0 neurons/mm^2^, *P* = 0.0005 for DNm2, and *P* = 0.0001 for DNm5) (Fig. 2A1–A4).

Protein kinase RNA-like ER kinase(+) neurons were expressed by small-diameter neurons (Fig. 2B1–B4), and these PERK(+) neurons were colocalized with Bcl-_XL_(+) neurons (Fig. 2C). Furthermore, the density changes of PERK(+) and Bcl-_XL_(+) neurons showed paralleled fashions (Fig. 2D). Statistically, the Manders coefficients of M1 Bcl-_XL_/PERK was 0.48 to 0.61 and M2 PERK/Bcl-_XL_ was 0.41 to 0.61 (Fig. 2E). Furthermore, simple linear regression analyses indicated that the Bcl-_XL_ and PERK neuron densities were inversely correlated with the thermal latency (Bcl-_XL_: *r* = −0.65, *P* = 0.0006 and PERK: *r* = −0.80, *P* < 0.0001; Fig. 2F and G) and the mechanical threshold (Bcl-_XL_: *r* = −0.72, *P* < 0.0001 and PERK: *r* = −0.69, *P* = 0.0002; Fig. 2H and I).

### 3.2. Coexpression of protein kinase RNA-like ER kinase(+):eukaryotic initiation factor 2α(+) neurons was correlated with neuropathic pain in diabetic neuropathy

**Figure 3.**
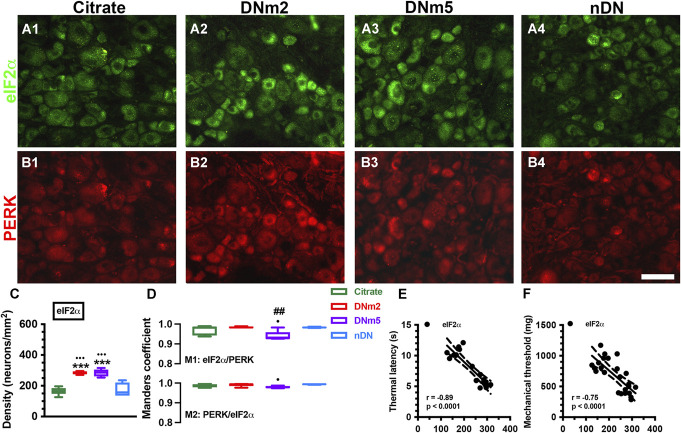
Expression patterns of phosphorylated eukaryotic initiation factor 2α (eIF2α) and phosphorylated protein kinase RNA-like ER kinase (PERK) in mice with streptozotocin (STZ)-induced diabetic neuropathy (DN). Double-labeling immunofluorescence staining was performed with anti-eIF2α (A1–A4, green) and anti-PERK (B1–B4, red) antisera in dorsal root ganglia in the citrate (A1 and B1; n = 6), DNm2 (A2 and B2; n = 5), DNm5 (A3 and B3; n = 5), and non-DN (nDN) groups (blood glucose <400 mg/dL; A4 and B4; n = 5). DNm2 and DNm5: mice with blood glucose >400 mg/dL at 2 and 5 months after receiving STZ, respectively. Bar, 50 µm. (C, D) Density changes in eIF2α(+) (left panel in C) and (D) Manders coefficients M1 (upper panel) and M2 (lower panel) of eIF2α(+):PERK(+) neurons were examined according to merged photos from A and B. eIF2α(+) neurons were upregulated at DNm2 and DNm5. ***P < 0.001: DNm2 or DNm5 vs the citrate group. •*P* < 0.05, •••*P* < 0.001: DNm2 or DNm5 vs the nDN group. ##*P* < 0.01: DNm2 vs the DNm5 group. (E, F) eIF2α(+) neuron densities were inversely correlated with the changes in the thermal latency (E) and mechanical threshold (F).

Protein kinase RNA-like ER kinase mediates ER stress and sequentially phosphorylates eIF2α. This study examined the coexpression profiles of PERK and phosphorylates eIF2α (Fig. 3). Eukaryotic initiation factor 2α was colocalized with PERK (Fig. 3A and B). For example, eIF2α(+) neuron densities were significantly increased at DNm2 (285.3 ± 10.5 vs 164.8 ± 23.9 neurons/mm^2^, *F* = 27.07, *P* < 0.0001) and DNm5 (284.1 ± 24.0 neurons/mm^2^, *P* < 0.0001) similar to PERK(+) neuronal profiles (Fig. 2D vs 3C). The Manders coefficients showed highly colocalized as follows: M1 eIF2α/PERK: 0.94 to 0.98 and M2 PERK/eIF2α: 0.97 to 0.98 (Fig. 3D). In addition, eIF2α(+) neuron densities were inversely correlated with the thermal latency (*r* = −0.89, *P* < 0.0001; Fig. 3E) and the mechanical threshold (*r* = −0.75, *P* < 0.0001; Fig. 3F).

### 3.3. Coexpression profiles of protein kinase RNA-like ER kinase(+):ubiquitin D(+) neurons in mice with diabetic neuropathy

**Figure 4.**
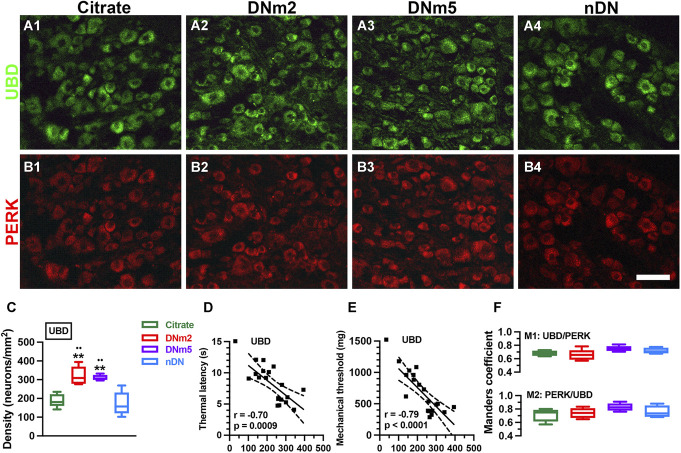
Colocalized patterns of ubiquitin D (UBD) and phosphorylated protein kinase RNA-like ER kinase (PERK) in mice with streptozotocin (STZ)-induced diabetic neuropathy (DN). Double-labeling immunofluorescence staining was performed with anti-UBD (A1–A4, green) and anti-PERK (B1–B4, red) antisera in the dorsal root ganglia in the citrate (A1 and B1; n = 5), DNm2 (A2 and B2; n = 5), DNm5 (A3 and B3; n = 5), and non-DN (nDN) groups (blood glucose <400 mg/dL; A4 and B4; n = 5). DNm2 and DNm5: mice with blood glucose >400 mg/dL at 2 and 5 months after receiving STZ, respectively. Bar, 50 µm. (C–E) Density changes in UBD(+) neurons are quantified (C) and were inverse linearly correlated with the changes in thermal latency (D) and mechanical threshold (E). **P < 0.01: DNm2 or DNm5 vs the citrate group. ••*P* < 0.01: DNm2 or DNm5 vs the nDN group. (D) Manders coefficients M1 (upper panel) and M2 (lower panel) of UBD(+):PERK(+) neurons were analyzed according to merged photos from A and B.

We then examined the neuromorphology of ubiquitination signaling by investigating the colocalization of UBD(+):PERK(+) neurons (Fig. 4). The coexpression of UBD(+) neurons with PERK(+) neurons was detected (Fig. 4A and B). Ubiquitin D(+) neuron densities were increased at DNm2 (320.7 ± 49.6 vs 189.3 ± 35.5 neurons/mm^2^, *F* = 15.44, *P* = 0.0013) and DNm5 (311.0 ± 14.7 neurons/mm^2^, *P* = 0.0027) compared with the citrate and nDN groups (172.4 ± 63.4 neurons/mm^2^; Fig. 4C). Ubiquitin D(+) neuron densities were also inversely correlated with the thermal latency (Fig. 4D) and the mechanical threshold (Fig. 4E). Moreover, Manders coefficient analyses indicated the high colocalization of UBD and PERK (Fig. 4F).

This study conducted neuromorphological examinations and demonstrated the relationships among the upregulation of Bcl-_XL_, ER stress, and ubiquitination signaling, which were all related to the development of neuropathic pain.

## 4. Discussion

### 4.1. Multiple roles of B-cell lymphoma-extra large in diabetic neuropathy

A previous study documented the neuromodulative effects of Bcl-_XL_, as indicated by, for example, the neuropathological profile of Bcl-_XL_ being correlated with neuropathic pain in DN. The upregulation of Bcl-_XL_ was injury dependent,^10^ suggesting that Bcl-_XL_ activation contributes to neuron injury. This observation implies that neuron injury induces ER stress. Although previous reports suggested that Bcl-_XL_ has neuroprotective properties,^9,12^ our series of studies further documented Bcl-_XL_ activation that mediates pain development.^10,19^ For example, PKCε modulates neuropathic pain,^3,27^ and our previous study indicated that the coexpression of PKCε(+):Bcl-_XL_(+) neuronal density was linearly related to the level of neuropathic pain.^10^ The expression profiles demonstrated that PKCε was coexpressed with ER stress–related signaling molecules.^21^ Moreover, Bcl-_XL_ was coexpressed with cytoplasmic polyadenylation element-binding protein, which is involved in protein translation and is essential for transmission by nociceptors.^6^ The deregulation of Bcl-_XL_ induced the apoptosis of pancreatic β cells.^24^ Conversely, Bcl-_XL_ overexpression exerted neuroprotective effects against the apoptosis of these β cells.^18,31^ Thus, apoptosis occurs through ER stress induction and UPS dysfunction.^24^ The study further documented the upregulation of Bcl-_XL_ and PERK, which mediate ER stress and ubiquitination signaling molecules, in the same nociceptors. This finding suggests that Bcl-_XL_ is a pivotal modulator of nerve injury and the intracellular neuroprotective mechanism by ER stress. Therefore, Bcl-_XL_ may be an upstream modulator that is involved in the modulation of ER stress signaling and DN development. The inactivation of Bcl-_XL_ expression in nociceptors is a potential target for relieving neuropathic pain in T1DM.

### 4.2. Correlation of endoplasmic reticulum stress and ubiquitination signaling with neuropathic pain in diabetic neuropathy

Functional proteins are essential for normal cellular physiology, and misfolded protein aggregates within a cell are removed through the protective mechanism of ER stress. Cophosphorylation of PERK:eIF2α is a signaling pathway that mediates ER stress. This study revealed the high colocalization of Bcl-_XL_(+) and PERK(+) nociceptors in DRG. In mice with DN, ER stress induction was particularly observed in injured nociceptors.^19^ Although the roles of Bcl-_XL_ in neuroprotection and of PERK signaling in ER stress have been widely addressed in epidemiologic studies on diabetes, no study has examined their coexpression profiles within the same cell, particularly in sensory nociceptors. This study is the first to provide neuropathological evidence for the correlation between ER stress signaling and the activities of molecules with neuroprotective effects. The neuropathological evidence revealed high M1 and M2 Manders coefficients for Bcl-_XL_:PERK. The upregulation of Bcl-_XL_(+) and PERK(+) neurons was inversely correlated with thermal latencies and mechanical thresholds. This finding suggests that these colocalized Bcl-_XL_(+):PERK(+) neurons contribute to neuropathic pain in T1DM. Notably, coimmunostaining approaches reveal the coexpression of interest protein/molecules within the same neurons, reflecting the pathological characteristics, but do not totally indicate the functional interactions. Therefore, further functional assay is required to clarify the signal regulation cascade.

Pathogenic misfolded proteins in the ER are degraded through ubiquitination and elimination by UPS.^25,26^ Ubiquitin-proteasome system is a vital process for balancing protein synthesis and degradation, and functional UPS requires several key enzymes and molecules, such as UBD, which is a key molecule in ubiquitination signaling involved in protein degradation. Our previous study revealed the correlation between ER stress and autophagy and pathological characteristics induced by misfolded protein accumulation. The present study revealed the correlation between ER stress and UPS activation that based on neuropathological evidence, which was confirmed by high M1 and M2 Manders coefficients for PERK:UBD coexpression.

### 4.3. Sex dimorphic features and clinical significance limitations in a male mouse type 1 diabetes mellitus model

Although a cohort study showed that both genders experienced similar pain frequently and neuropathic symptoms.^2^ However, male diabetic patients are more likely to suffer from nerve damage^5,7^ and early onset of peripheral neuropathy.^1^ The reports also indicated that female individuals experienced a higher pain intensity^2^ and higher diabetic-associated cardiac autonomic neuropathy.^4^ These diabetic complication features in females might be affected by hormone effects^29^ and the different degrees of small-fiber dysfunction^33^ in females. These mechanisms require further investigation into the mechanisms of pain cognition.^16^ In addition, the use of von Frey monofilament could explain the changes of mechanical threshold by tactile application, which reflects the alteration of static tactile reflexes, but does not represent the real painful sensation. Notably, these pathological data in this T1DM model with male mice are limited, and females might have more complicated pathophysiological neuromechanisms.

Collectively, inactivating Bcl-_XL_ might be a treatment strategy for neuropathic pain in T1DM, and the results jointly indicate that in DN, Bcl-_XL_ might be an upstream modulator that regulates the dynamic balance of ER stress and UPS activity for maintaining functional and physiological protein activity.

## Disclosures

The authors have no conflicts of interest to declare.

## Supplemental digital content

Supplemental digital content associated with this article can be found online at http://links.lww.com/PR9/A353.
